# SOX‐1 antibodies positive Lambert–Eaton myasthenic syndrome with occult small cell lung cancer: A case report

**DOI:** 10.1111/crj.13740

**Published:** 2024-03-18

**Authors:** Liming Zhao, Hongyan He, Weixin Han, Yizhe Meng, Lifei Kang, Yanqiang Chen

**Affiliations:** ^1^ Department of Neurology Hebei Chest Hospital Shijiazhuang Hebei China; ^2^ Department of Pathology Hebei Chest Hospital Shijiazhuang Hebei China

**Keywords:** Lambert–Eaton myasthenic syndrome, occult cancer, small cell lung cancer, SOX‐1 antibody

## Abstract

Lambert–Eaton myasthenic syndrome (LEMS) is a rare paraneoplastic neurological syndrome of the neuromuscular transmission. The symptoms often progress slowly and can be misdiagnosed in early stage. Seropositive SOX‐1 antibodies are support for the diagnosis of LEMS and have high specificity for small cell lung cancer (SCLC). In this paper, we report a case of a 56‐year‐old man with smoking history who was admitted to hospital with progressive muscle weakness of the proximal legs. LEMS was diagnosed by repetitive nerve stimulation (RNS) testing and seropositive SOX‐1 antibodies. Primary screening with chest computed tomography (CT) and integrated PET/CT did not reveal any tumor. After continuous follow‐up, SCLC was found by chest CT and confirmed with pathological examination 10 months after the diagnosis of LEMS. Long‐term follow‐up and screening for occult SCLC in LEMS patients with positive SOX‐1 antibodies are very important.

## Graphical abstract

We report a patient with LEMS who was tested positive for SOX‐1 antibodies and was definitively diagnosed with SCLC by pathological biopsy after 10 months follow‐up.

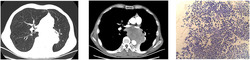

## 1 INTRODUCTION

Lambert–Eaton myasthenic syndrome (LEMS) is a rare autoimmune neuromuscular junction disorder.^1^, ^2^ The main features are muscle weakness of proximal lower limbs and general fatigue, which seriously affect the quality of life of patients.^3^, ^4^, ^5^

An estimated 50–60% of patients with LEMS are associated with tumors, especially small cell lung cancer (SCLC).^1^, ^3^ The pathogenic mechanism is that the antibodies directed against voltage‐gated calcium channels (VGCCs) expressed on the surface of tumor cells cross‐react with the VGCCs on presynaptic membrane, affecting neuromuscular transmission.^1^, ^6^

In recent years, the detection of antibodies has improved the diagnosis of LEMS. A series of studies have shown that SOX‐1 antibodies, initially called anti‐glial nuclear antibodies (AGNA), are associated with LEMS and specifically found in SCLC.^7^, ^8^, ^9^, ^10^ The SOX‐1 antibodies are found in 65% of patients with SCLC‐LEMS, 36.5% of patients with SCLC.^8^, ^9^

Here, we report a patient with LEMS who was tested seropositive for SOX‐1 antibodies and was definitively diagnosed with SCLC by pathological biopsy after 10 months follow‐up.

## 2 CASE REPORT

A 56‐year‐old man was admitted to our hospital with progressive muscle weakness of the lower limbs. He described symptoms as beginning 3 months before, with a sensation of heaviness in the proximal legs and skeletal muscle fatigue when walking. The patient had a medical history of rheumatoid arthritis and tuberculosis, and he previously smoked cigarettes.

His neurological examination revealed proximal muscles weakness of both legs (Medical Research Council Scale for Muscle Strength: 4), depressed deep tendon reflexes and dry mouth. Repetitive nerve stimulation (RNS) testing of the left abductor digiti minimi muscle elicited a mild decremental response (−7%) at low frequency (3 Hz) but an incremental response (>500%) at high frequency (20 Hz), confirming the presence of LEMS.^1^, ^4^

Serum antibodies related to neuromuscular junction disorders including muscle‐specific tyrosine kinase antibody, low‐density lipoprotein receptor‐related protein 4 antibody, acetylcholine receptor antibody and VGCC antibody were negative. However, the anti‐SOX‐1 antibody was positive, highly suggested of paraneoplastic LEMS. Well characterized onconeural antibodies including Hu‐Ab, Yo‐Ab, Ri‐Ab and Ma2‐Ab all tested negative.

**FIGURE 1:**
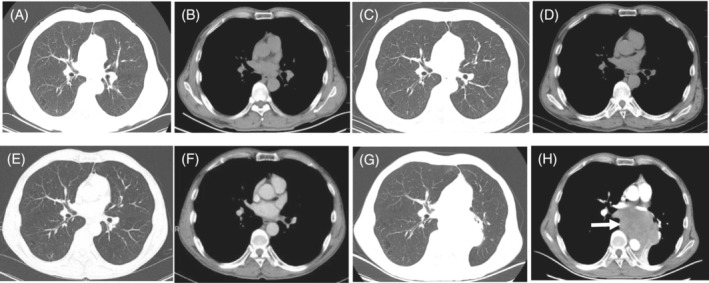
Dynamic changes in chest CT during admission and follow‐up. Chest CT at the time of diagnosis with LEMS (A, B). 3 months (C, D), 6 months (E, F) after the diagnosis of LEMS. Chest CT (G) and enhanced chest CT (H) 10 months after the diagnosis of LEMS demonstrated lung cancer of posterior inferior mediastinum and left pulmonary hilum (as indicated by the arrow).

Magnetic resonance imaging (MRI) in brain showed no brain metastases. The chest computed tomography (CT) revealed multiple solid nodules with calcification in bilateral lung caused by tuberculosis but uncovered any malignancies (Figure 1A,B). The integrated positron emission tomography and computed tomography (PET/CT) scan for malignancy was negative. The patient was also screened by bronchoscopy, but no tumor cells was found.

Combination with prednisone and azathioprine was applied for the long‐term treatment. The follow‐up thoracic CT for tumor was negative at 3 months (Figure 1C,D) and 6 months (Figure 1E,F) after the diagnosis of LEMS.

**FIGURE 2:**
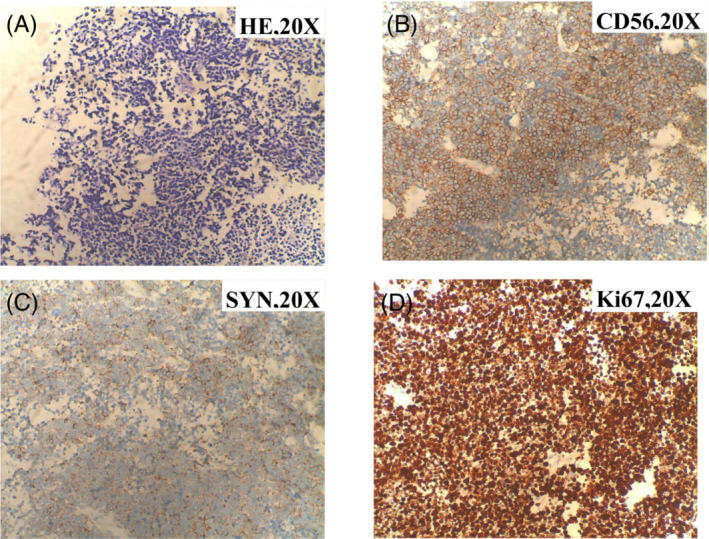
The histological and immunohistochemical analysis on pathological slides was consistent with SCLC.^11^ Hematoxylin and eosin staining showed that small cells with diffuse patchy growth were circular or elliptical in shape, with scant cytoplasm, finely granular nuclear chromatin and inconspicuous nucleoli (A). Immunohistochemical stain showed that cells were positive for CD56 (B) and synaptophysin (C), and the proliferation index Ki67 was about 90% (D). SCLC: small cell lung cancer, CD: cluster of differentiation.

The patient admitted to our hospital with a chief complaint of shortness of breath after activity 10 months later. However, the strength of the lower limbs did not become worsen compared to his first hospitalization. Thoracic CT showed a huge soft tissue mass of posterior inferior mediastinum and left pulmonary hilum, which was inhomogeneous enhancement in enhanced chest CT (Figure 1G,H). A subsequent CT guided needle biopsy of the mass was performed that revealed pathological findings of SCLC (Figure 2). Because SCLC was in the advanced stage, the patient was discharged and received chemotherapy in local hospital.

## 3 DISCUSSION

The main clinical manifestation of LEMS is usually characterized by proximal muscle weakness, reduced or absent tendon reflexes and dysautonomia.^1^, ^2^, ^5^ Proximal muscle weakness in the legs is usually the first symptom noted by the patient. Autonomic dysfunction is another common symptom including dry mouth, erectile dysfunction in men and constipation.^2^, ^4^

In recent years, P/Q‐type VGCC antibodies are found in 85–90% of patients with LEMS.^2^, ^3^ However, VGCC antibodies was seronegative in this patient. Studies showed that passive transfer the autoantibodies from patients with negative for P/Q‐type VGCC antibodies to mice also induces disease.^12^ The possible explanations have been entertained because the antibodies have subthreshold concentration or antibodies to different epitopes of VGCC are not recognized.^3^, ^6^

The SOX‐1 protein is developmental transcription factors and thought to prevent neural differentiation in progenitor cells.^7^, ^9^ The protein is important in airway epithelial differentiation, which do not express in normal bronchial epithelium cells.^7^ SOX‐1 antibodies are associated with various paraneoplastic syndromes especially LEMS and classified as biomarkers of SCLC.^7^, ^8^, ^9^, ^13^ The SOX‐1 antibodies have 67% sensitivity and 95% specificity to discriminate between SCLC‐LEMS and nontumor LEMS.^8^

Symptoms of LEMS occur before diagnosis of SCLC in the majority of patients.^5^, ^14^, ^15^ Previous studies showed that SCLC was detected in 91% within 3 months and in 96% within 1 year after the diagnosis of LEMS.^14^ In this patient, we performed thorax CT, bronchoscopy, and integrated PET/CT to screen for SCLC when LEMS was diagnosed. Because the first screening for tumor was negative, continuously follow‐up was performed as mentioned. SCLC was diagnosed 10 months later with the histopathology of needle biopsy of mass.

In SCLC‐associated paraneoplastic syndrome, Guillain–Barre syndrome (GBS) should be account for differential diagnosis of LEMS.^16^ Both diseases have clinical manifestations of limb weakness and decreased reflexes. Weakness of LEMS normally spreads proximally to distally. On the contrary, weakness of GBS spreads distally to proximally. GBS usually have sensory symptoms by contrast with LEMS. Nerve conduction studies of GBS mainly show decrease in nerve conduction velocity and reduction in compound muscle action potential (CMAP) amplitude.^17^ RNS of LEMS show increase in the CMAP amplitude of at least 100% at high frequency (20–50 Hz) stimulation.^4^

LEMS is a rare disease often accompanied by tumors especially SCLC. Antibodies tests are important for LEMS and SOX‐1 antibodies usually indicate the presence of SCLC. When the diagnosis of LEMS is made, we should initiate tumor screening immediately. If primary screening is negative, a long period of clinical follow‐up and screening for an underlying tumor should be performed.

## AUTHOR CONTRIBUTIONS

Liming Zhao and Hongyan He retrieved literatures and wrote the manuscript. Weixin Han and Yizhe Meng collected data and revised the manuscript. Lifei Kang provided pathological findings. Liming Zhao and Yanqiang Chen reviewed and approved the final version before submission. All authors have read and approved the final manuscript.

## CONFLICT OF INTEREST STATEMENT

All authors declare that they have no conflict of interest.

## ETHICS STATEMENT

This study was approved by the Ethics Committee of Hebei Chest Hospital. The patient provided written informed consent before inclusion into this study.

## Data Availability

The data that support the findings of this study are available from the corresponding author upon reasonable request.
